# Age differences in head motion and estimates of cortical morphology

**DOI:** 10.7717/peerj.5176

**Published:** 2018-07-27

**Authors:** Christopher R. Madan

**Affiliations:** School of Psychology, University of Nottingham, Nottingham, United Kingdom

**Keywords:** Head motion, Cortical structure, Fractal dimensionality, Age, Cortical thickness, Gyrification, Cortical morphology, Movie watching, BMI

## Abstract

Cortical morphology is known to differ with age, as measured by cortical thickness, fractal dimensionality, and gyrification. However, head motion during MRI scanning has been shown to influence estimates of cortical thickness as well as increase with age. Studies have also found task-related differences in head motion and relationships between body–mass index (BMI) and head motion. Here I replicated these prior findings, as well as several others, within a large, open-access dataset (Centre for Ageing and Neuroscience, CamCAN). This is a larger dataset than these results have been demonstrated previously, within a sample size of more than 600 adults across the adult lifespan. While replicating prior findings is important, demonstrating these key findings concurrently also provides an opportunity for additional related analyses: critically, I test for the influence of head motion on cortical fractal dimensionality and gyrification; effects were statistically significant in some cases, but small in magnitude.

## Introduction

Head motion during the acquisition of magnetic resonance imaging (MRI) can lead to artifacts when estimating brain activity and structure. With functional MRI (fMRI), volumes are acquired relatively quickly–often every 1–3 s–allowing for the estimation and correction of head motion artifacts. Using innovative techniques such as prospective motion correction (Dosenbach et al., 2017; Federau & Gallichan, 2016; Maclaren et al., 2013; Stucht et al., 2015; Tisdall et al., 2016) and custom-designed, individualized head-cases (https://caseforge.co), effects of head motion can be attenuated. However, these solutions are not suitable for large studies of inter-individual differences in brain morphology where changes to the MRI scan sequence or custom-built equipment for each participant are often not practical. In the current study, I assessed relationships between age and body–mass index (BMI) on head motion, task-related differences in head motion, and the influence of head motion on estimates of cortical morphology. In light of these findings, many of which are replications, I propose a potential method for attenuating head motion during structural MRIs, as well as discuss limitations of this method.

Prior studies have demonstrated that older adults tend to have more head motion than younger adults (Andrews-Hanna et al., 2007; Chan et al., 2014; Savalia et al., 2017; Pardoe, Hiess & Kuzniecky, 2016). Unfortunately, other studies have also provided evidence that head motion can lead to lower cortical thickness estimates (Alexander-Bloch et al., 2016; Pardoe, Hiess & Kuzniecky, 2016; Reuter et al., 2015; Savalia et al., 2017), as such, age-related differences in cortical thickness (e.g., Fjell et al., 2009; McKay et al., 2014; Salat et al., 2004) may be exaggerated by age-related differences in head motion. In addition to age, obesity has also been associated with head motion (Beyer et al., 2017; Hodgson et al., 2017). In particular, these associations have been shown with respect to body–mass index (BMI; kg/m^2^), which is measured as body weight (in kg) divided by body height (in m) squared–despite the relatively coarse nature of BMI (e.g., does not differentiate between muscle vs. fat mass) (Diverse Populations Collaborative Group, 2005; Romero-Corral et al., 2008). Findings of relationships between obesity and cortical thickness have been mixed (Shaw et al., 2017; Shaw et al., 2018; Veit et al., 2014). More generally, head motion has been suggested to be a neurobiological trait–being both stable over time and heritable (Engelhardt et al., 2017; Hodgson et al., 2017; Zeng et al., 2014).

There is also evidence that fMRI tasks can differ in the degree of associated head motion (Alexander et al., 2017; Huijbers et al., 2017; Greene et al., 2018; Vanderwal et al., 2015; Wylie et al., 2014; Yuan et al., 2009). With this in mind, it may be beneficial to present participants with a task to attend to *during structural scans*, with the objective of decreasing head motion; typically structural scans are accompanied by the presentation of a blank screen or otherwise lack of instruction of attending to a visual stimulus.

Madan & Kensinger (2016) showed that a structural metric, fractal dimensionality (FD), may be more sensitive to age-related differences in cortical structure than cortical thickness (also see Madan & Kensinger, 2018). In a preliminary analysis to examine the influence of head motion on age-related differences in cortical fractal dimensionality, Madan & Kensinger (2016) showed qualitative evidence of age-related differences in fractal dimensionality in a small sample (*N* = 7) of post-mortem MRIs. However, as this sample was small and also less indicative of potential head motion effects in *in vivo* MR imaging, further work is necessary. To more directly test for the additive influence of head motion on estimates of cortical morphology, beyond aging, here I also tested for a relationship of fMRI-estimated head motion on cortical fractal dimensionality, as well as on mean cortical thickness. Additionally, as recent studies have found that gyrification also decreases with age (Cao et al., 2017; Hogstrom et al., 2013; Madan & Kensinger, 2016; Madan & Kensinger, 2018), it was also included in the analysis presented here. Test-retest reliability of estimates for these structural measures has recently been compared (Madan & Kensinger, 2017b), but robustness to head motion has yet to be assessed.

Using the rich, open-access dataset from Cambridge Centre for Ageing and Neuroscience (CamCAN) (Shafto et al., 2014; Taylor et al., 2017), here I sought to replicate these myriad of prior findings, as well as test for influences of head motion on fractal dimensionality and gyrification.

## Methods

### Dataset

Data used in the preparation of this work were obtained from the Cambridge Centre for Ageing and Neuroscience (CamCAN) repository, available at http://www.mrc-cbu.cam.ac.uk/datasets/camcan/ (Shafto et al., 2014; Taylor et al., 2017). The CamCAN dataset includes structural and functional MRI data for a sample of 648 adults across the adult lifespan (aged 18–88; Mean (SD) =54.2(18.5)). All participants were cognitively healthy (MMSE >24) and were free of any neurological or serious psychiatric conditions. See Shafto et al. (2014) for additional details about the sample inclusion and exclusion criteria.

A total of eight participants were excluded from further analyses due to problems with cortical reconstruction or gyrification estimation, yielding a final sample size of 640 adults (326 female, 314 male). Height and weight measurements were available for 559 of the 648 participants (280 female, 279 male), additionally allowing for the calculation of body–mass index (BMI) for this subset of participants (also see Ronan et al., 2016).

Structural measures are derived from a T1-weighted volume acquired using a 3 T Siemens Trio MRI scanner with an MPRAGE sequence. Scan parameters were as follows: TR  = 2,250 ms, TE  = 2.99 ms, flip angle  = 9°, voxel size  = 1 × 1 × 1 mm, GRAPPA  = 2, TI  = 900 ms. Head motion was primarily estimated from two fMRI scans, during rest and a movie-watching task. Both scans lasted for 8 min and 40 s (i.e., 520 s total). For the rest scan, participants were instructed to rest with their eyes closed. For the movie scan, participants watched and listened to condensed version of Alfred Hitchcock’s (1961) “Bang! You’re Dead” (Campbell et al., 2015; Hasson et al., 2008). Note that different scan sequences were used for both of these scans, with volumes collected every 1.970 s or 2.470 s for the rest and movie scans, respectively (see Taylor et al., 2017 for more details); both rest and movie scans had the same voxel size, 3 × 3 × 4.44 mm (32 axial slices, 3.7 mm thick, 0.74 mm gap).

### Preprocessing of the structural MRI data

The T1-weighted structural MRIs were processed using FreeSurfer v6.0 (https://surfer.nmr.mgh.harvard.edu/) (Dale, Fischl & Sereno, 1999; Fischl, 2012; Fischl & Dale, 2000). Surface meshes and cortical thickness was estimated using the standard processing pipeline, i.e., recon-all, and no manual edits were made to the surfaces. Gyrification was calculated using FreeSurfer, as described in Schaer et al. (2012).

Fractal dimensionality (FD) is a measure of the complexity of a structure and has previously been shown to decrease in relation to aging for cortical (Madan & Kensinger, 2016; Madan & Kensinger, 2018) and subcortical (Madan & Kensinger, 2017a; Madan, 2018) structures and has been shown to have high test-retest reliability (Madan & Kensinger, 2017b). FD was calculated using the calcFD toolbox (http://cmadan.github.io/calcFD/) (Madan & Kensinger, 2016) using the dilation method and filled structures (denoted as *FD*_*f*_ in prior studies). Briefly, FD measures the effective dimensionality of a structure by counting how many grid ‘boxes’ of a particular size are needed to contain a structure; these counts are then contrasted relative to the box sizes in log-space, yielding a scale-invariant measure of the complexity of a structure. This is mathematically calculated as *FD* =  − Δlog_2_(Count)∕Δlog_2_(Size), where Size was set to {1, 2, 4, 8, 16} (i.e., powers of 2, ranging from 0 to 4). To correct for the variability in FD estimates associated with the alignment of the box-grid with the structure, a dilation algorithm was used which instead relies on a 3D-convolution operation (convn in MATLAB) as this approach yields more reliable estimates of FD. This computational issue is described mathematical and demonstrated in simulations in Madan & Kensinger (2016), and empirically shown in Madan & Kensinger (2017b). See Madan & Kensinger (2016) and Madan & Kensinger (2018) for additional background on fractal dimensionality and its application to brain imaging data.

### Estimates of head motion

Head motion was estimated using two approaches:

(1) Measured as the frame-wise displacement using the three translational and three rotational realignment parameters. Realignment parameters were included as part of the preprocessed fMRI data (Taylor et al., 2017), in the form of the rp_*.txt output generated by the SPM realignment procedure. Rotational displacements were converted from degrees to millimeters by calculating the displacement on the surface of a sphere with a radius of 50 mm (as in Power et al., 2012). Frame-wise displacement was substantially higher between volumes at the beginning of each scan run, so the first five volumes were excluded. This is the same approach to estimating head motion that is commonly used (e.g., Alexander-Bloch et al., 2016; Engelhardt et al., 2017; Power et al., 2012; Savalia et al., 2017).

(2) Estimated directly from the T1-weighted volume as ‘average edge strength’ (AES) (Aksoy et al., 2012; Zacà et al., in press). This approach measures the intensity of contrast at edges within an image. Higher AES values correspond to less motion, with image blurring yielding decreased tissue contrast. AES was calculated using the toolbox provided by Zacà et al. (in press), on the skull-stripped volumes generated as an intermediate stage of the FreeSurfer processing pipeline. AES is calculated on two-dimensional image planes and was performed on each plane orientation (axial, sagittal, and coronal).

### Model comparison approach

Effects of head motion on estimates of cortical morphology (thickness, fractal dimensionality, and gyrification) were assessed using a hierarchical regression procedure using MATLAB. Age was first input, followed by BMI (both with and without age), followed by estimates of head motion from each fMRI scan and the related interaction term with age. In total, eight models were examined, as listed in Table 1. Model fitness was assessed using both *R*^2^ and Δ*BIC*.

Bayesian Information Criterion, *BIC*, is a model fitness index that includes a penalty based on the number of free parameters (Schwarz, 1978). Smaller *BIC* values correspond to better model fits. By convention, two models are considered equivalent if Δ*BIC* < 2 (Burnham & Anderson, 2004). As *BIC* values are based on the relevant dependent variable, Δ*BIC* values are reported relative to the best-performing model (i.e.,  Δ*BIC* = 0 for the best model considered).

**Table 1:** Variance explained and model fits of cortical measures by age, BMI, and head motion estimates. Note that *R*^2^ decreases after the inclusion of BMI as models 2 and 3 can only be calculated on a subset of participants (559 out of 640 participants) since height and weight information was not available for all participants.

		Thickness		FD		Gyrification	
Model	Predictors	*R*^2^	ΔBIC	*R*^2^	ΔBIC	*R*^2^	ΔBIC
1	Age	.425	6.98	.497	3.15	.192	3.65
2	BMI	.029	455.85	.028	805.25	.007	243.17
3	Age + BMI	.425	168.82	.487	454.69	.183	140.23
4	Age + Movement(Rest)	.429	10.01	.500	5.65	.192	10.07
5	Age + Movement(Movie)	.437	**0.00**	.504	**0.00**	.194	8.44
6	Age + Movement(Movie) + Age × Movement (Movie)	.427	11.64	.499	6.58	.205	**0.00**
7	Age + AES(axial)	.443	**0.23**	.507	3.18	.194	14.83
8	Age + AES(axial) + Age × AES(axial)	.428	17.58	.500	12.42	.208	3.76

## Results

### fMRI-estimated head motion

**Figure 1:**
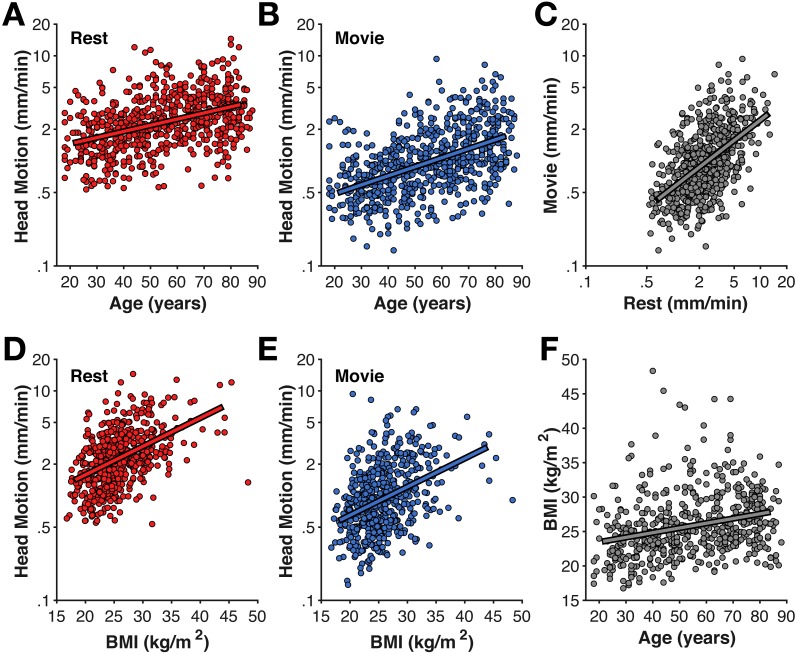
Age-related differences in head motion. Correlations between average head motion (mm/min) with age for the (A) rest and (B) movie fMRI scans, with (D–E) body–mass index (BMI), (C) between fMRI scans, and (F) between age and BMI. Head motion axes are log-10 scaled to better show inter-individual variability.

As shown in Fig. 1, older adults head increased head motion relative to younger adults in both the rest and movie scans (rest: *r*(638) = .351, *p* < .001; movie: *r*(638) = .430, *p* < .001). Head motion was also greater in the rest scan than during the movie watching (*t*(639) = 23.35, *p* < .001, Cohen’s *d* = 0.99, *M*_*diff*_ = 1.528 mm/min). Nonetheless, head motion was correlated between the fMRI scans [*r*(638) = .484, *p* < .001]. While this correlation between scans is expected, particularly since both were collected in the same MRI session, studies have provided evidence that head motion during scanning may be a trait (Engelhardt et al., 2017; Hodgson et al., 2017; Zeng et al., 2014). Moreover, this correlation provides additional evidence that motion during the fMRI scans is consistently larger in some individuals than others, suggesting it similarly affected the structural scans more for some individuals than others and appropriate to include as a predictor for the cortical morphology estimates.

As expected based on prior literature (Beyer et al., 2017; Hodgson et al., 2017), head motion was also correlated with body–mass index (BMI) (rest: *r*(557) = .456, *p* < .001; movie: *r*(557) = .335, *p* < .001) (Fig. 1). While BMI was also correlated with age (*r*(557) = .274, *p* < .001), BMI-effects on head motion persisted after accounting for age differences (rest: *r*_*p*_(555) = .340, *p* < .001; movie: *r*_*p*_(555) = .249, *p* < .001).

**Figure 2:**
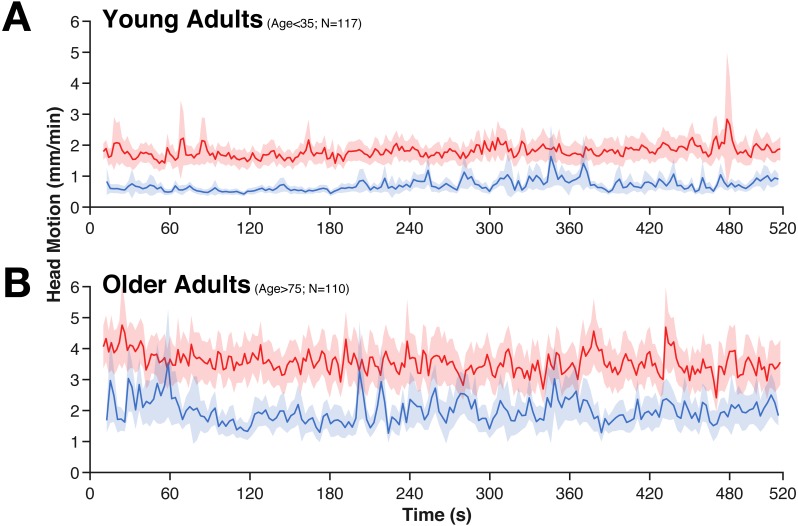
Averaged time-course of head motion for rest (red) and movie (blue) fMRI scans for young (A) and older adults (B). Bands represent 95% confidence intervals.

While head motion was substantially lower in the movie condition than during rest, it was relatively stable over time (e.g., it does not tend to decrease over time). However, in the movie watching task, there is evidence of systematic stimuli-evoked increases in head motion (Fig. 2), e.g., around 280 s and 360 s. These periods of increased head motion correspond to events within the movie; in the first period, the boy is loading the real gun with bullets, the second, more prominent period is a suspenseful scene where it appears that the boy may accidentally shoot someone. Moreover, these events also correspond to fMRI differences in attentional control and inter-subject synchrony (see Campbell et al., 2015).

**Figure 3:**
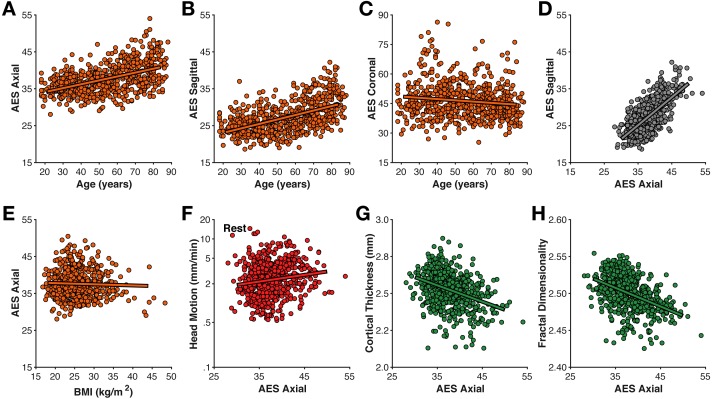
Relationships between motion estimated from the structural volume using average edge strength (AES) in (A–C) different planes with age, (D) between planes, (E) BMI, (F) rest-fMRI estimated motion, (G) cortical thickness, and (H) fractal dimensionality.

### T1-estimated head motion

Head motion was also estimated directly from the T1-weighted volume as the average edge strength (AES), following from Zacà et al. (in press); higher AES values correspond to less motion. Here I calculated AES for each plane orientation. AES in the axial and sagittal planes was moderately related to age (axial: *r*(639) = .493, *p* < .001; sagittal: *r*(639) = .525, *p* < .001) (Fig. 3); AES in the coronal was only weakly correlated with age (*r*(639) =  − .131, *p* < .001). AES in the axial and sagittal planes were strongly correlated with each other (*r*(639) = .702, *p* < .001).

Interestingly, AES was relatively not related to BMI (all |*r*|’s <.2). AES was also relatively unrelated to fMRI-estimated head motion (rest: *r*(639) = .112, *p* = .005; movie: *r*(639) = .148, *p* < .001). Thus, while AES is sensitive to an MR image property related to age, it seems to be distinct from fMRI-estimated head motion. A likely possibility is that AES here is detecting age-related differences in gray/white matter contrast ratio (GWR), as have been previously observed (Knight et al., 2016; Magnaldi et al., 1993; Salat et al., 2009). In contrast, the mechanism for the correlation between BMI and fMRI-estimated head motion is likely apparent–rather than real–head motion caused by respiratory chest motion producing susceptibility variations in the B0 field (Raj, Anderson & Gore, 2001; Van de Moortele et al., 2002; Van Gelderen et al., 2007).

**Figure 4:**
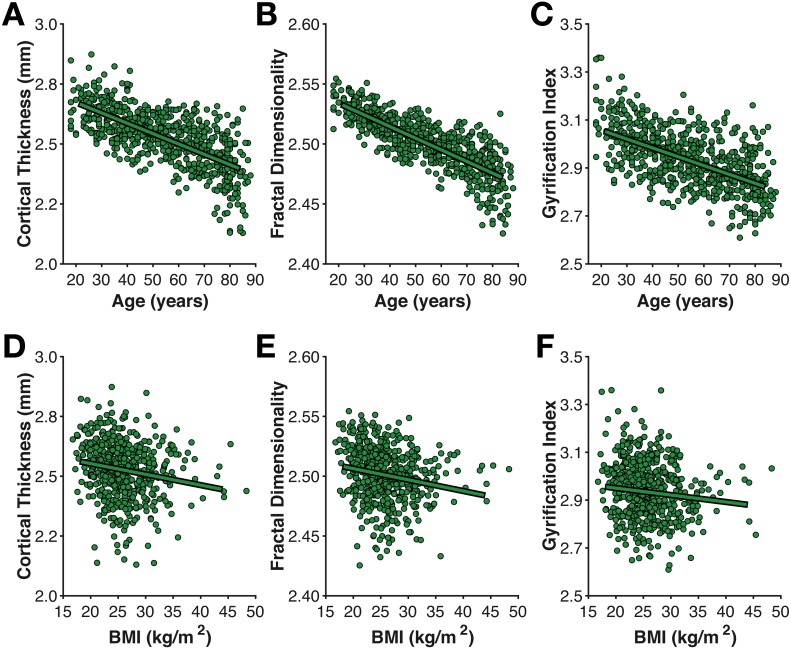
Age- and BMI-related differences in the three cortical morphology measures examined here: (A, D) thickness, (B, E) fractal dimensionality, and (C, F) gyrification.

### Cortical morphology

As shown in Fig. 4, mean cortical thickness significantly decreased with age (*r*(638) =  − .652, *p* < .001, −0.0432 mm/decade), as did fractal dimensionality (*r*(638) =  − .705, *p* < .001, −0.0097 *FD*_*f*_/decade) and gyrification (*r*(638) =  − .427, *p* < .001, −0.0372 *GI*/decade). All three slopes (change in metric per decade) are nearly identical to those first calculated by Madan & Kensinger (2016), as is the finding of higher age-related differences in fractal dimensionality and weaker differences in gyrification (also see Madan & Kensinger, 2018). However, it is also worth acknowledging that AES in the axial and sagittal planes were comparably correlated with age as gyrification. Effects of BMI on all three measures of cortical morphology were relatively weak (thickness: *r*(557) =  − .169, *p* < .001; fractal dimensionality: *r*(557) =  − .168, *p* < .001; gyrification *r*(557) =  − .083, *p* = .049).

Of particular interest, I examined the influence of head motion on the cortical morphology estimates. For all three measures, head motion explained only a small amount of additional variance beyond age, as shown in Table 1. Nonetheless, head motion from the movie scan did explain significant additional variance, as measured by ΔBIC, however, this only accounted for an additional 1% variance in the cortical morphology measures. In the model of cortical thickness including head motion from the movie scan (but not the interaction), age related changes corresponded to −0.0398 mm/decade, while head motion contributed −0.0135 mm/(mm/min).

## Discussion

In the current study, I replicated several prior findings as well as tested for a few novel effects of head motion. First I outline the key findings of prior studies that were replicated here:

(1) Increased head motion in older adults (replicating Savalia et al., 2017; Pardoe, Hiess & Kuzniecky, 2016).
(2) BMI is correlated with fMRI-estimated head motion (replicating Beyer et al., 2017; Hodgson et al., 2017).
(3) Less head motion occurs when watching a movie than during rest (replicating Vanderwal et al., 2015; Huijbers et al., 2017).
(4) Head motion in different scans from the same individuals is correlated and indexes reliable inter-individual differences (replicating Zeng et al., 2014; Engelhardt et al., 2017; Hodgson et al., 2017).
(5) Cortical thickness decreases with age (replicating Fjell et al., 2009; Salat et al., 2004).
(6) Fractal dimensionality and gyrification also decrease with age (replicating Madan & Kensinger, 2016; Madan & Kensinger, 2018; Hogstrom et al., 2013).
(7) More head motion leads to lower estimates of cortical thickness (replicating Reuter et al., 2015; Savalia et al., 2017).

In addition to these replications, the new findings were:

(8) Head motion leads to nominally lower estimates of fractal dimensionality and gyrification.
(9) Head motion estimated from the structural volume itself (i.e., average edge strength [AES]) correlated with age, but not BMI.
(10) AES may be sensitive to gray/white matter contrast ratio (GWR).
(11) AES was only weakly related to fMRI-estimated head motion.
(12) Global cortical morphology is weakly related to BMI.

Likely most important, I found significantly more movement during resting state than watching a movie, but are quite correlated still (replicating the findings of Huijbers et al., 2017; Greene et al., 2018). Based on this evidence, I would recommend that participants be given movie-watching task *during structural scans* to reduce movement during these longer volume acquisitions and improve scan quality. Suggestions of potential systematic increases in head motion, however, suggest that less eventful movie content may be preferable for both maintaining participants’ attention and minimizing movement-based reactions (e.g., see Vanderwal et al., 2015). While this approach is not common, it has been used in some recent large-scale studies, such as the Human Connectome Project (HCP) (Marcus et al., 2013) and Adolescent Brain Cognitive Development (ABCD) study (Casey et al., in press), and has also been suggested and used elsewhere, particularly in MRI studies with children (Greene, Black & Schlaggar, 2016; De Bellis et al., 2001; Howell et al., in press; Overmeyer, 1996; Pliszka et al., 2006; Raschle et al., 2009; Theys, Wouters & Ghesquière, 2014; Von Rhein et al., 2015; Wu Nordahl et al., 2008). However, it is also important to consider the context that this movie watching would occur in. For instance, if the structural scan is followed by a resting-state fMRI scan, cognitive processes related to the movie watching will ‘spill over’ and influence patterns of brain activity in a subsequent rest period (e.g., Tambini & Davachi, 2013; Van Kesteren et al., 2010; Eryilmaz et al., 2011).

Estimates of cortical thickness were significantly influenced by head motion (replicating Savalia et al., 2017; Reuter et al., 2015), though the influence of this appeared to be relatively small. Effects of head motion on fractal dimensionality were also significant, but even smaller in magnitude, while head motion did not significantly influence estimates of gyrification. The results here also served as a replication age-related differences in fractal dimensionality and gyrification (Madan & Kensinger, 2016; Madan & Kensinger, 2018).

Interestingly, average edge strength (AES) did not correlate well with fMRI-estimated motion, but did correlate with age. This may be related to age-related differences in gray/white matter contrast ratio (GWR), as AES corresponds to the degree of tissue intensity contrast. This finding may be important when examining differences in AES between different samples (e.g., patients vs. controls).

While the results here are predominately replications of prior work, they nonetheless integrate the key findings of several papers through a single, open-access dataset, that also has a larger sample size than these previous studies. Moreover, these results serve as an example to highlight the benefits of open data sharing on improving our understanding of brain morphology (see Madan, 2017 for a detailed discussion).

## Conclusion

Head motion influences estimates of cortical morphology, but can be attenuated by using an engaging task, such as movie watching, rather than merely instructing participants to rest. Decreasing head motion is particularly important when studying aging populations, where head motion is greater than for young adults, but considerations are necessary to see how this may ‘carry over’ and influence a subsequent scan, such as resting-state fMRI.

##  Supplemental Information

10.7717/peerj.5176/supp-1 Supplemental Information 1 Derived brain morphology measures from CamCAN data Each row corresponds to one participant. The “mvmt” columns correspond to estimated head motion in the rest and movie-watching fMRI scans. “ct”, “gyrif”, and “fd” correspond to estimated cortical morphology measures: cortical thickness, gyrification, and fractal dimensionality. The “aes” columns correspond to the AES measure in the axial, coronal, and sagittal planes, respectively. BMI is the body-mass index.

## Additional Information and Declarations

### Data Availability

The following information was supplied regarding data availability: The unprocessed T1 structural data, head-motion regressors from the processed resting-state and movie-watching functional MRI data, along with demographic (age, sex) and physical (height, weight) data are available at Cam-CAN Data Portal
https://camcan-archive.mrc-cbu.cam.ac.uk/dataaccess/. The measures derived in this article’s analysis are available here: Christopher R. Madan. (2018). Derived brain morphology measures from CamCAN data [Data set]. Zenodo. http://doi.org/10.5281/zenodo.1258016.

## References

[ref-1] Aksoy M, Forman C, Straka M, Çukur T, Hornegger J, Bammer R (2012). Hybrid prospective and retrospective head motion correction to mitigate cross-calibration errors. Magnetic Resonance in Medicine.

[ref-2] Alexander LM, Escalera J, Ai L, Andreotti C, Febre K, Mangone A, Vega-Potler N, Langer N, Alexander A, Kovacs M, Litke S, O’Hagan B, Andersen J, Bronstein B, Bui A, Bushey M, Butler H, Castagna V, Camacho N, Chan E, Citera D, Clucas J, Cohen S, Dufek S, Eaves M, Fradera B, Gardner J, Grant-Villegas N, Green G, Gregory C, Hart E, Harris S, Horton M, Kahn D, Kabotyanski K, Karmel B, Kelly SP, Kleinman K, Koo B, Kramer E, Lennon E, Lord C, Mantello G, Margolis A, Merikangas KR, Milham J, Minniti G, Neuhaus R, Levine A, Osman Y, Parra LC, Pugh KR, Racanello A, Restrepo A, Saltzman T, Septimus B, Tobe R, Waltz R, Williams A, Yeo A, Castellanos FX, Klein A, Paus T, Leventhal BL, Craddock RC, Koplewicz HS, Milham MP (2017). An open resource for transdiagnostic research in pediatric mental health and learning disorders. Scientific Data.

[ref-3] Alexander-Bloch A, Clasen L, Stockman M, Ronan L, Lalonde F, Giedd J, Raznahan A (2016). Subtle in-scanner motion biases automated measurement of brain anatomy from in vivo MRI. Human Brain Mapping.

[ref-4] Andrews-Hanna JR, Snyder AZ, Vincent JL, Lustig C, Head D, Raichle ME, Buckner RL (2007). Disruption of large-scale brain systems in advanced aging. Neuron.

[ref-5] Beyer F, Masouleh SK, Huntenburg JM, Lampe L, Luck T, Riedel-Heller SG, Loeffler M, Schroeter ML, Stumvoll M, Villringer A, Witte AV (2017). Higher body mass index is associated with reduced posterior default mode connectivity in older adults. Human Brain Mapping.

[ref-6] Burnham KP, Anderson DR (2004). Multimodel inference. Sociological Methods & Research.

[ref-7] Campbell KL, Shafto MA, Wright P, Tsvetanov KA, Geerligs L, Cusack R, Tyler LK, Tyler LK, Brayne C, Bullmore E, Calder A, Cusack R, Dalgleish T, Duncan J, Henson R, Matthews F, Marslen-Wilson W, Rowe J, Shafto M, Campbell K, Cheung T, Davis S, Geerligs L, Kievit R, McCarrey A, Price D, Taylor J, Tsvetanov K, Williams N, Bates L, Emery T, Erzinçlioglu S, Gadie A, Gerbase S, Georgieva S, Hanley C, Parkin B, Troy D, Allen J, Amery G, Amunts L, Barcroft A, Castle A, Dias C, Dowrick J, Fair M, Fisher H, Goulding A, Grewal A, Hale G, Hilton A, Johnson F, Johnston P, Kavanagh-Williamson T, Kwasniewska M, McMinn A, Norman K, Penrose J, Roby F, Rowland D, Sargeant J, Squire M, Stevens B, Stoddart A, Stone C, Thompson T, Yazlik O, Dixon M, Barnes D, Hillman J, Mitchell J, Villis L (2015). Idiosyncratic responding during movie-watching predicted by age differences in attentional control. Neurobiology of Aging.

[ref-8] Cao B, Mwangi B, Passos IC, Wu M-J, Keser Z, Zunta-Soares GB, Xu D, Hasan KM, Soares JC (2017). Lifespan gyrification trajectories of human brain in healthy individuals and patients with major psychiatric disorders. Scientific Reports.

[ref-9] Casey B, Cannonier T, Conley MI, Cohen AO, Barch DM, Heitzeg MM, Soules ME, Teslovich T, Dellarco DV, Garavan H, Orr CA, Wager TD, Banich MT, Speer NK, Sutherland MT, Riedel MC, Dick AS, Bjork JM, Thomas KM, Chaarani B, Mejia MH, Hagler DJ, Cornejo MD, Sicat CS, Harms MP, Dosenbach NU, Rosenberg M, Earl E, Bartsch H, Watts R, Polimeni JR, Kuperman JM, Fair DA, Dale AM The Adolescent Brain Cognitive Development (ABCD) study: imaging acquisition across 21 sites. Developmental Cognitive Neuroscience.

[ref-10] Chan MY, Park DC, Savalia NK, Petersen SE, Wig GS (2014). Decreased segregation of brain systems across the healthy adult lifespan. Proceedings of the National Academy of Sciences of the United States of America.

[ref-11] Dale AM, Fischl B, Sereno MI (1999). Cortical surface-based analysis: I. Segmentation and surface reconstruction. NeuroImage.

[ref-12] De Bellis MD, Keshavan MS, Beers SR, Hall J, Frustaci K, Masalehdan A, Noll J, Boring AM (2001). Sex differences in brain maturation during childhood and adolescence. Cerebral Cortex.

[ref-13] Diverse Populations Collaborative Group (2005). Weight-height relationships and body mass index: some observations from the diverse populations collaboration. American Journal of Physical Anthropology.

[ref-14] Dosenbach NU, Koller JM, Earl EA, Miranda-Dominguez O, Klein RL, Van AN, Snyder AZ, Nagel BJ, Nigg JT, Nguyen AL, Wesevich V, Greene DJ, Fair DA (2017). Real-time motion analytics during brain MRI improve data quality and reduce costs. NeuroImage.

[ref-15] Engelhardt LE, Roe MA, Juranek J, DeMaster D, Harden KP, Tucker-Drob EM, Church JA (2017). Children’s head motion during fMRI tasks is heritable and stable over time. Developmental Cognitive Neuroscience.

[ref-16] Eryilmaz H, Ville DVD, Schwartz S, Vuilleumier P (2011). Impact of transient emotions on functional connectivity during subsequent resting state: a wavelet correlation approach. NeuroImage.

[ref-17] Federau C, Gallichan D (2016). Motion-correction enabled ultra-high resolution in-vivo 7 T-MRI of the brain. PLOS ONE.

[ref-18] Fischl B (2012). FreeSurfer. NeuroImage.

[ref-19] Fischl B, Dale AM (2000). Measuring the thickness of the human cerebral cortex from magnetic resonance images. Proceedings of the National Academy of Sciences of the United States of America.

[ref-20] Fjell AM, Westlye LT, Amlien I, Espeseth T, Reinvang I, Raz N, Agartz I, Salat DH, Greve DN, Fischl B, Dale AM, Walhovd KB (2009). High consistency of regional cortical thinning in aging across multiple samples. Cerebral Cortex.

[ref-21] Greene DJ, Black KJ, Schlaggar BL (2016). Considerations for MRI study design and implementation in pediatric and clinical populations. Developmental Cognitive Neuroscience.

[ref-22] Greene DJ, Koller JM, Hampton JM, Wesevich V, Van AN, Nguyen AL, Hoyt CR, McIntyre L, Earl EA, Klein RL, Shimony JS, Petersen SE, Schlaggar BL, Fair DA, Dosenbach NU (2018). Behavioral interventions for reducing head motion during MRI scans in children. NeuroImage.

[ref-23] Hasson U, Landesman O, Knappmeyer B, Vallines I, Rubin N, Heeger DJ (2008). Neurocinematics: the neuroscience of film. Projections.

[ref-24] Hitchcock A (1961). Bang! You’re Dead [Motion Picture].

[ref-25] Hodgson K, Poldrack RA, Curran JE, Knowles EE, Mathias S, Gring HH, Yao N, Olvera RL, Fox PT, Almasy L, Duggirala R, Barch DM, Blangero J, Glahn DC (2017). Shared genetic factors influence head motion during MRI and body mass index. Cerebral Cortex.

[ref-26] Hogstrom LJ, Westlye LT, Walhovd KB, Fjell AM (2013). The structure of the cerebral cortex across adult life: age-related patterns of surface area, thickness, and gyrification. Cerebral Cortex.

[ref-27] Howell BR, Styner MA, Gao W, Yap P-T, Wang L, Baluyot K, Yacoub E, Chen G, Potts T, Salzwedel A, Li G, Gilmore JH, Piven J, Smith JK, Shen D, Ugurbil K, Zhu H, Lin W, Elison JT The UNC/UMN baby connectome project (BCP): an overview of the study design and protocol development. NeuroImage.

[ref-28] Huijbers W, Van Dijk KRA, Boenniger MM, Stirnberg R, Breteler MMB (2017). Less head motion during MRI under task than resting-state conditions. NeuroImage.

[ref-29] Knight MJ, McCann B, Tsivos D, Couthard E, Kauppinen RA (2016). Quantitative T1 and T2 MRI signal characteristics in the human brain: different patterns of MR contrasts in normal ageing. Magnetic Resonance Materials in Physics, Biology and Medicine.

[ref-30] Maclaren J, Herbst M, Speck O, Zaitsev M (2013). Prospective motion correction in brain imaging: a review. Magnetic Resonance in Medicine.

[ref-31] Madan CR (2017). Advances in studying brain morphology: the benefits of open-access data. Frontiers in Human Neuroscience.

[ref-32] Madan CR (2018). Shape-related characteristics of age-related differences in subcortical structures. Aging & Mental Health.

[ref-33] Madan CR, Kensinger EA (2016). Cortical complexity as a measure of age-related brain atrophy. NeuroImage.

[ref-34] Madan CR, Kensinger EA (2017a). Age-related differences in the structural complexity of subcortical and ventricular structures. Neurobiology of Aging.

[ref-35] Madan CR, Kensinger EA (2017b). Test–retest reliability of brain morphology estimates. Brain Informatics.

[ref-36] Madan CR, Kensinger EA (2018). Predicting age from cortical structure across the lifespan. European Journal of Neuroscience.

[ref-37] Magnaldi S, Ukmar M, Vasciaveo A, Longo R, Pozzi-Mucelli R (1993). Contrast between white and grey matter: MRI appearance with ageing. European Radiology.

[ref-38] Marcus DS, Harms MP, Snyder AZ, Jenkinson M, Wilson JA, Glasser MF, Barch DM, Archie KA, Burgess GC, Ramaratnam M, Hodge M, Horton W, Herrick R, Olsen T, McKay M, House M, Hileman M, Reid E, Harwell J, Coalson T, Schindler J, Elam JS, Curtiss SW, Essen D. CV (2013). Human Connectome Project informatics: quality control, database services, and data visualization. NeuroImage.

[ref-39] McKay DR, Knowles E. EM, Winkler A. AM, Sprooten E, Kochunov P, Olvera RL, Curran JE, Kent JW, Carless MA, Göring HHH, Dyer TD, Duggirala R, Almasy L, Fox PT, Blangero J, Glahn DC (2014). Influence of age, sex and genetic factors on the human brain. Brain Imaging and Behavior.

[ref-40] Overmeyer S (1996). Angstverarbeitung von psychisch aufflligen Kindern im Kernspintomogramm. Monatsschrift Kinderheilkunde.

[ref-41] Pardoe HR, Hiess RK, Kuzniecky R (2016). Motion and morphometry in clinical and nonclinical populations. NeuroImage.

[ref-42] Pliszka SR, Lancaster J, Liotti M, Semrud-Clikeman M (2006). Volumetric MRI differences in treatment-naive vs chronically treated children with ADHD. Neurology.

[ref-43] Power JD, Barnes KA, Snyder AZ, Schlaggar BL, Petersen SE (2012). Spurious but systematic correlations in functional connectivity MRI networks arise from subject motion. NeuroImage.

[ref-44] Raj D, Anderson AW, Gore JC (2001). Respiratory effects in human functional magnetic resonance imaging due to bulk susceptibility changes. Physics in Medicine and Biology.

[ref-45] Raschle NM, Lee M, Buechler R, Christodoulou JA, Chang M, Vakil M, Stering PL, Gaab N (2009). Making MR imaging child’s play—pediatric neuroimaging protocol, guidelines and procedure. Journal of Visualized Experiments.

[ref-46] Reuter M, Tisdall MD, Qureshi A, Buckner RL, Van der Kouwe AJ, Fischl B (2015). Head motion during MRI acquisition reduces gray matter volume and thickness estimates. NeuroImage.

[ref-47] Romero-Corral A, Somers VK, Sierra-Johnson J, Thomas RJ, Collazo-Clavell ML, Korinek J, Allison TG, Batsis JA, Sert-Kuniyoshi FH, Lopez-Jimenez F (2008). Accuracy of body mass index in diagnosing obesity in the adult general population. International Journal of Obesity.

[ref-48] Ronan L, Alexander-Bloch AF, Wagstyl K, Farooqi S, Brayne C, Tyler LK, Fletcher PC (2016). Obesity associated with increased brain age from midlife. Neurobiology of Aging.

[ref-49] Salat DH, Buckner RL, Snyder AZ, Greve DN, Desikan R. SR, Busa E, Morris JC, Dale AM, Fischl B (2004). Thinning of the cerebral cortex in aging. Cerebral Cortex.

[ref-50] Salat DH, Lee SY, Van der Kouwe AJ, Greve DN, Fischl B, Rosas HD (2009). Age-associated alterations in cortical gray and white matter signal intensity and gray to white matter contrast. NeuroImage.

[ref-51] Savalia NK, Agres PF, Chan MY, Feczko EJ, Kennedy KM, Wig GS (2017). Motion-related artifacts in structural brain images revealed with independent estimates of in-scanner head motion. Human Brain Mapping.

[ref-52] Schaer M, Cuadra MB, Schmansky N, Fischl B, Thiran J-P, Eliez S (2012). How to measure cortical folding from MR images: a step-by-step tutorial to compute local gyrification index. Journal of Visualized Experiments.

[ref-53] Schwarz G (1978). Estimating the dimension of a model. Annals of Statistics.

[ref-54] Shafto MA, Tyler LK, Dixon M, Taylor JR, Rowe JB, Cusack R, Calder AJ, Marslen-Wilson WD, Duncan J, Dalgleish T, Henson RN, Brayne C, Matthews FE (2014). The Cambridge Centre for Ageing and Neuroscience (Cam-CAN) study protocol: a cross-sectional, lifespan, multidisciplinary examination of healthy cognitive ageing. BMC Neurology.

[ref-55] Shaw ME, Abhayaratna WP, Anstey KJ, Cherbuin N (2017). Increasing body mass index at midlife is associated with increased cortical thinning in Alzheimer’s disease-vulnerable regions. Journal of Alzheimer’s Disease.

[ref-56] Shaw ME, Sachdev PS, Abhayaratna W, Anstey KJ, Cherbuin N (2018). Body mass index is associated with cortical thinning with different patterns in mid- and late-life. International Journal of Obesity.

[ref-57] Stucht D, Danishad KA, Schulze P, Godenschweger F, Zaitsev M, Speck O (2015). Highest resolution in vivo human brain MRI using prospective motion correction. PLOS ONE.

[ref-58] Tambini A, Davachi L (2013). Persistence of hippocampal multivoxel patterns into postencoding rest is related to memory. Proceedings of the National Academy of Sciences of the United States of America.

[ref-59] Taylor JR, Williams N, Cusack R, Auer T, Shafto MA, Dixon M, Tyler LK, Henson RN, Cam-CAN (2017). The Cambridge Centre for Ageing and Neuroscience (Cam-CAN) data repository: structural and functional MRI, MEG, and cognitive data from a cross-sectional adult lifespan sample. NeuroImage.

[ref-60] Theys C, Wouters J, Ghesquière P (2014). Diffusion tensor imaging and resting-state functional MRI-scanning in 5- and 6-year-old children: training protocol and motion assessment. PLOS ONE.

[ref-61] Tisdall MD, Reuter M, Qureshi A, Buckner RL, Fischl B, Van der Kouwe AJ (2016). Prospective motion correction with volumetric navigators (vNavs) reduces the bias and variance in brain morphometry induced by subject motion. NeuroImage.

[ref-62] Van Kesteren MTR, Fernandez G, Norris DG, Hermans EJ (2010). Persistent schema-dependent hippocampal-neocortical connectivity during memory encoding and postencoding rest in humans. Proceedings of the National Academy of Sciences of the United States of America.

[ref-63] Vanderwal T, Kelly C, Eilbott J, Mayes LC, Castellanos FX (2015). Inscapes: a movie paradigm to improve compliance in functional magnetic resonance imaging. NeuroImage.

[ref-64] Van de Moortele P, Pfueffer J, Glover GH, Ugurbil K, Hu X (2002). Respiration-induced B0 fluctuations and their spatial distribution in the human brain at 7 Tesla. Magnetic Resonance in Medicine.

[ref-65] Van Gelderen P, De Zwart JA, Starewicz P, Hinks RS, Duyn JH (2007). Real-time shimming to compensate for respiration-induced B0 fluctuations. Magnetic Resonance in Medicine.

[ref-66] Veit R, Kullmann S, Heni M, Machann J, Häring H-U, Fritsche A, Preissl H (2014). Reduced cortical thickness associated with visceral fat and BMI. NeuroImage: Clinical.

[ref-67] Von Rhein D, Mennes M, Van Ewijk H, Groenman AP, Zwiers MP, Oosterlaan J, Heslenfeld D, Franke B, Hoekstra PJ, Faraone SV, Hartman C, Buitelaar J (2015). The NeuroIMAGE study: a prospective phenotypic, cognitive, genetic and MRI study in children with attention-deficit/hyperactivity disorder. Design and descriptives. European Child & Adolescent Psychiatry.

[ref-68] Wu Nordahl C, Simon TJ, Zierhut C, Solomon M, Rogers SJ, Amaral DG (2008). Methods for acquiring structural MRI data in very young children with autism without the use of sedation. Journal of Autism and Developmental Disorders.

[ref-69] Wylie GR, Genova H, DeLuca J, Chiaravalloti N, Sumowski JF (2014). Functional magnetic resonance imaging movers and shakers: does subject-movement cause sampling bias?. Human Brain Mapping.

[ref-70] Yuan W, Altaye M, Ret J, Schmithorst V, Byars AW, Plante E, Holland SK (2009). Quantification of head motion in children during various fMRI language tasks. Human Brain Mapping.

[ref-71] Zacà D, Hasson U, Minati L, Jovicich J Method for retrospective estimation of natural head movement during structural MRI. Journal of Magnetic Resonance Imaging.

[ref-72] Zeng L-L, Wang D, Fox MD, Sabuncu M, Hu D, Ge M, Buckner RL, Liu H (2014). Neurobiological basis of head motion in brain imaging. Proceedings of the National Academy of Sciences of the United States of America.

